# High-copy bacterial plasmids diffuse in the nucleoid-free space, replicate stochastically and are randomly partitioned at cell division

**DOI:** 10.1093/nar/gkt918

**Published:** 2013-10-16

**Authors:** Rodrigo Reyes-Lamothe, Tung Tran, Diane Meas, Laura Lee, Alice M. Li, David J. Sherratt, Marcelo E. Tolmasky

**Affiliations:** ^1^Department of Biochemistry, University of Oxford, Oxford OX1 3QU, UK, ^2^Department of Biology, McGill University, Montreal, Quebec H3G 0B1, Canada and ^3^Department of Biological Science, Center for Applied Biotechnology Studies, College of Natural Science and Mathematics, California State University Fullerton, Fullerton, CA 92834-6850, USA

## Abstract

Bacterial plasmids play important roles in the metabolism, pathogenesis and bacterial evolution and are highly versatile biotechnological tools. Stable inheritance of plasmids depends on their autonomous replication and efficient partition to daughter cells at cell division. Active partition systems have not been identified for high-copy number plasmids, and it has been generally believed that they are partitioned randomly at cell division. Nevertheless, direct evidence for the cellular location of replicating and nonreplicating plasmids, and the partition mechanism has been lacking. We used as model pJHCMW1, a plasmid isolated from *Klebsiella pneumoniae* that includes two β-lactamase and two aminoglycoside resistance genes. Here we report that individual ColE1-type plasmid molecules are mobile and tend to be excluded from the nucleoid, mainly localizing at the cell poles but occasionally moving between poles along the long axis of the cell. As a consequence, at the moment of cell division, most plasmid molecules are located at the poles, resulting in efficient random partition to the daughter cells. Complete replication of individual molecules occurred stochastically and independently in the nucleoid-free space throughout the cell cycle, with a constant probability of initiation per plasmid.

## INTRODUCTION

Plasmids play an essential role in bacterial metabolism, pathogenesis and evolution by harboring genes coding for diverse factors and facilitating their dissemination. Of particular importance is their role in the dissemination of resistance genes contributing to the current epidemic of resistant infections (1,2). Several mechanisms, including regulation of initiation of replication, partition, multimer resolution and post-segregational killing, ensure that plasmids are stably maintained at a constant copy number within the host bacterial cells and are transmitted to the following generations (3,4). To ensure their segregation, low copy number plasmids code for mechanisms that actively mediate the partition of the molecules among daughter cells (3). Conversely, high copy number (hcn) plasmids, which are usually smaller than low copy number plasmids, appear not to code for partition systems and their stable inheritance must be assured by the high number of copies of the plasmid molecules. The lack of partition systems led to the idea that they segregate by diffusing randomly through the cytoplasm, and the high number of copies was enough to ensure that every daughter cell receives at least one plasmid molecule (5,6). Later, the implementation of fluorescence microscopy in bacteria showed fewer spots per cell than those expected of fluorescently labeled plasmids, leading to the suggestion that hcn plasmids are not randomly located and may be bound or clustered together (3,7–10). These intriguing results prompted us to further study the segregation and replication of the clinically relevant ColE1-type plasmid pJHCMW1 using quantitative imaging methods. This hcn plasmid was first identified in a *Klebsiella pneumoniae* strain isolated from a neonate with meningitis during a nosocomial infection and is responsible for failure of treatment by conferring resistance to several antibiotics (11).

To track its position in the cell, we inserted a cassette containing a *tetO* operator array (12) within a location unrelated to replication or maintenance. Our results suggest that the plasmid’s preferred localization reflects its inability to migrate through nucleoid-dense regions, a property most probably common to all plasmids. The plasmid molecules were nevertheless highly mobile and seemed to migrate as independent units. pJHCMW1 replicated stochastically during the cell cycle, and plasmid replication events were distributed randomly through the population of cells. Our results provide an explanation to the apparent contradiction between the genetic suggestions of random diffusion and the localized clustering inferred previously from microscopy experiments.

## MATERIALS AND METHODS

### Construction of plasmids and strains

Operator arrays were obtained by digesting the plasmids from the pLAU series (12) with *Xba*I and *Nhe*I, and were ligated to *Avr*II-digested pJHCMW1 DNA, creating pTT1 (24 operators), pTT3 (48 operators) and pTT4 (96 operators). A fluorescent version of *tetR* regulated by a *lac* promoter was introduced into the chromosome by λ Red recombination replacing *galK* (13). Replisome components were labeled with YPet as described before (13,14). The *dnaA46*(ts) allele was used to prevent replication of the chromosome. A deletion of *yjjG* was used to increase the incorporation of EdU (15). A strain carrying the *dnaN159*(ts) allele was used to produce elongated cells with a high nucleoid-free volume. All strains used in this study are derivatives of AB1157 with exception of strain ES1 carrying the temperature-sensitive DNA polymerase I gene *polA12*(ts) [F-, *lacY1 or lacZ53*(Am), *uvrC34*, *rpsL31*(strR) *or rpsL151*(strR), *polA12*(ts)]. Diameter of gyration of pJHCMW1 was calculated from reported experimental data for other plasmids in their supercoiled form (16,17).

### Microscopy

Bacterial cells were cultured in M9-glycerol, where the generation time is ∼100 min at 37°C. When used, cephalexin was used at a concentration of 40 µg/ml to stop cell division. Cells were collected at OD_600_ 0.1–0.2 and spotted in 1% agarose pads for microscopy. Time-lapses were done using M9-glycerol agarose pads, and slides were incubated at least 30 min and kept at 30°C for the length of the experiment. Cells were visualized with a 100× objective on a Nikon Eclipse TE2000-U microscope, equipped with a Photometrics Cool-SNAP HQ CCD camera. For the observation of pTT3 and pTT4, we used capture times of up to 1 or 2 s for time-lapse and snapshot experiments. Analysis was done using ImageJ or the Matlab base MicrobeTracker (18,19).

### Intracellular dilution of plasmids using *polA12*

*Escherichia coli* ES1 cells carrying the *tetR-yfp* cassette in their chromosomes and pTT4 were grown overnight in Luria Broth (LB) at 30°C under the selection of ampicillin to maintain the plasmid. They were then diluted into fresh medium without antibiotics and incubated at 42°C for at least four generation times (∼2 h) before spotting them on an agarose pad with M9-Glucose + 1/20 dilution of LB. The slide was incubated at the microscope at 40°C to prevent re-initiation of the plasmid replication. Use of M9-glycerol for this experiment was not possible because of the unexpected halt to cell growth at restrictive conditions when plasmid was present.

### Particle tracking and determination of confinement area

Automatic particle tracking was done using View5D application in ImageJ. In those cases where the particle became too dim for the program to detect it, we used a manual guided mode for the detection of the missing steps. Cell boundaries were detected manually in ImageJ. Data were then processed in Matlab. To determine the asymptote of 1D mean square displacement (MSD) curves, to assign a particle confinement area, we fitted the curves using the following equation:

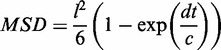

where 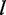 represents the confinement. This was obtained from an approximation of the equation described by Kusumi *et al.* (20).

To obtain an estimate of the apparent diffusion coefficient (D_app_) from the experimental data described in Derman *et al.* (21), we used their reported mean positional change of 0.20 ± 0.12 µm/5 s for the mini-RK2 plasmid MP924. We obtained the MSD from this number and used the formula D_app_ = MSD/(τ *q_i_) to calculate the D_app_. Here τ is the time between intervals and q_i_ is 4 given that the measurements were done in two dimensions.

### EdU labeling

*Escherichia coli dna46* cells carrying plasmids were cultured in M9-glycerol until early exponential phase (OD_600_ ∼0.1), shifted to 42°C for 2 h and incubated with 100 µg/ml deoxydenosine for 30 min. Nucleotide analogue EdU was added to cultures at a concentration of 20 µg/ml and cells were collected at different time points mixing them with chilled methanol for a final concentration of 70%. Cells were then pelleted and washed with phosphate buffered saline (PBS) three times, and then resuspended in 50 µl of the Click-iT reaction cocktail (Invitrogen), containing 20 µM Alexa Fluor 594 Azide, and incubated for 30 min at room temperature. Cells were washed three times more with PBS before observation. To rule out low efficiency of incorporation of EdU in newly synthesized plasmids, SSB-YPet–labeled strains carrying pJHCMW1 were used in time-lapse experiments. Wild-type or *dnaA46*(ts) cells were incubated at 32°C and 42°C, respectively. The number of plasmid replication events was determined by counting the number of polar spots in the first time point that disappeared in subsequent frames. A total of 821 and 581 cells were counted for the wild-type and the mutant strains, respectively.

### qPCR

Strains used for qPCR were cultured in M9-glycerol at 30°C until OD_600_ 0.1–0.2, then 200 μl were collected and the samples were heated at 94°C for 10 min and then frozen at −20°C to disrupt the cells without considerably affecting the ratio chromosome to plasmid (22). Samples were then thawed and diluted 100-fold. Chromosomal and plasmid DNA were diluted 100-fold as well. qPCR SYBR Green Mix (2×) (Bioline) was mixed with 0.8 μM of forward and reverse primers complementary to either the chromosomal DNA or plasmid DNA and sample or control DNA. As control DNA, we used chromosomal DNA extracted from *E. coli* AB1157 and pTT4 DNA.

Primers were complementary to noncoding regions near *oriC* at the chromosome and close to the β-lactamase gene in pJHCMW1. Primers used were Q_dws_gidB_F (5′-tgaagcacgctttatcacca-3′) and Q_dws_gidB_R (5′-gcatcaaaaagcggtcaaat-3′), and QpTT_1F (5′-ggaaccactccgttaggaca-3′) and QpTT_1R (5′-cgccacagattatgcaaaga-3′) for the chromosome and plasmid, respectively.

Amplification and measurement was done using a Corbett Rotor Gene 6000 qPCR machine, using 50 cycles of 95°C for 7 s, 59°C for 12 s and 68°C for 12 s. Analysis was done using Corbett LifeScience CAS-1200. Average number of plasmids was obtained by comparing the number of plasmids relative to the number of copies of *oriC* region of the chromosome. Using data from previous studies on the timing of replication, we estimated that in the growth conditions used, there is an average number of 1.9 *oriC* per cell (13,23). A slightly higher number than the one described in the results was obtained by dividing the total number of plasmids in the sample by the number of colony forming units calculated using the OD_600_ of the culture. This strategy resulted in an average of 2.6 (±0.3) *oriCs* and 24 (±7) pJHCMW1s per cell.

To account for the differences in cell cycle, we derived the following equation:

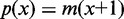

where *p* is the number of plasmids, *x* is the stage in the cell cycle and *m* is 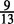 *k*. *k* is the average number of plasmids per volume of culture. When a cell is newly born, *x* = 0, resulting in ∼12 plasmids per cell. When a cell is about to divide, *x* = 1, resulting in ∼24 plasmids per cell. As a result, there are differing numbers of plasmids depending on what stage in the cell cycle the cell is in.

## RESULTS

### pJHCMW1 molecules accumulate preferentially at the poles

We used a Fluorescent Repressor Operator System (FROS) to detect the position of derivatives of plasmid pJHCMW1 inside live *E. coli* cells. A plasmid derivative containing 48 or 96 copies of *tetO* inserted into the *tnpA* gene was introduced into an *E. coli* strain in which fluorescent Tet repressor was expressed at a low level from a chromosomal *tetR* gene (Supplementary Figure S1A and B). The plasmid derivatives were stably inherited over 70 generations regardless of the presence of the repressor (Supplementary Figure S1C).

**Figure 1.**
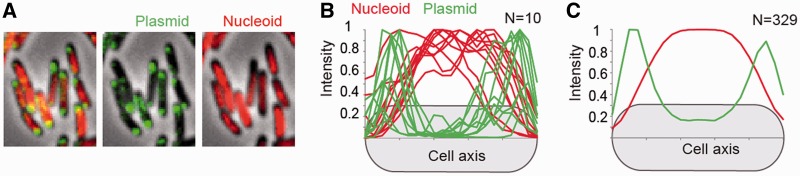
Localization of plasmid pJHCMW1. (**A**) Representative images of cells carrying the pJHCMW1 derivatives pTT3 and pTT4 labeled using TetR-YPet. The image to the left shows an overlay of plasmid and nucleoid signals, this latter labeled with Fis-mCherry. Middle and right pictures show the signal for the plasmid and nucleoid, respectively. (**B**) Plot showing 10 normalized fluorescent signal traces of plasmid (green) and nucleoid (red) over the cell length. Short cells were chosen in which splitting of sister nucleoids was not detected. (**C**) Average of the normalized fluorescent signal traces for 329 cells. The size of the cells considered was within the same range as those in (B).

The majority of the fluorescence was localized at the poles, an observation that agrees with previous reports on the intracellular distribution of hcn plasmids (7,9,10) (Figure 1A–C). Localization of the plasmid coincided with nucleoid-free regions as assessed by a fluorescent derivative of the DNA-binding protein Fis (Figure 1A–C). To understand whether localization was determined by a specific mechanism or just occupation of nucleoid-free space, we used mutant cells in which the free-nucleoid volume was increased and observed that plasmids occupied the increased space (Supplementary Figure S1D). Furthermore, in cells treated with the cell division inhibitor cephalexin that grew as long filaments with evenly spaced nucleoids, the plasmids accumulated not only at the poles but also in the spaces between these nucleoids (Supplementary Figure S1E). Hence, we inferred that the plasmids occupy the nucleoid-free space rather than being targeted to the poles by an active mechanism, reminiscent to the exclusion suffered by ribosomes, whose diameter of gyration of 18 nm (24) is significantly smaller than that calculated for the supercoiled 11.3-kb pJHCMW1 plasmid of 265 nm (‘Materials and Methods’ section).

### Continuous plasmid movement is restricted by the nucleoid

A fraction of plasmids did not localize to nucleoid-free regions, but instead were observed as small smears over the nucleoid. Time-lapse analysis, using few seconds intervals, showed plasmids moving, sometimes covering distances approaching the length of the cell in few frames (Video S1). Plasmids at the poles, normally visualized as a blob, also seemed to move since their fluorescent pattern changed shape over time (Video S1). Study of the kinetics of plasmid movement was nevertheless hindered by the density of plasmid molecules in the cell, estimated at an average of 17 [±4 standard deviation (SD)] copies per cell by qPCR (‘Materials and Methods’ section). Therefore, we reduced the plasmid copy number using a PolI_ts_ strain, since DNA replication of ColE1-type plasmids is initiated by PolI before being handed over to a PolIII replisome for completion (25–27). Shift to nonpermissive temperature blocked plasmid synthesis, while chromosome replication continued. Maintaining this condition over five generation times resulted in an uneven distribution of plasmids in cells, with many of them having no fluorescent spots and some having few. Each spot was considered a single plasmid molecule, although we cannot rule out the possibility that some spots represented plasmid dimers or catenanes. The spots were brighter than those observed in wild-type cells, likely due to a higher number of repressor molecules bound to individual plasmids. The intensity of these spots was also heterogeneous, presumably reflecting different levels of the repressor in cells. In our experiments, a small number of spots had high intensity and showed almost no movement, and we considered them as artifacts, although their nature was not clear to us.

**Figure 2.**
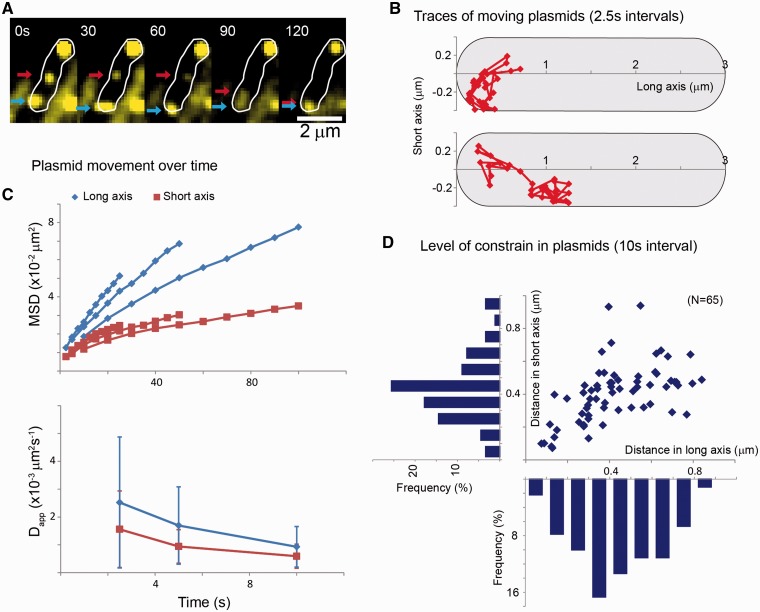
Plasmid dynamics. (**A**) Time-lapse at 30-s intervals of a *polA*(ts) cell containing three independent plasmid spots after diluting plasmids increasing the temperature of incubation. Red arrow shows a plasmid spot that moves over half of the cell length in 30 s. The blue arrow shows a spot that remains at the pole for the length of the movie. (**B**) Independent traces of the movement of two plasmid spots, one remaining at the pole and one moving away from it (top and bottom, respectively). (**C**) Plot for the MSD (top) and the apparent diffussion rate (D_app_, bottom) of traces obtained at 2.5 (N = 74), 5 (N = 79) and 10 s (N = 90). Error bars represent SD. (**D**) Projected area of constraint in the cell for individual plasmids studied at 10-s intervals obtained from their MSD curves.

We measured the rate of plasmid movement in intervals of 2.5, 5 and 10 s. Some of the spots moved away from the poles in the duration of the experiment, but most localized at the cell poles and remained there (Figure 2A and B). MSD of the plasmid was determined with respect to the long and short axis of the cell. We found movement over the long axis to be greater, similar to studies of chromosomal loci (Figure 2C) (13,28). Plotting the MSD over different intervals of time also showed a concave-shaped curve characteristic of sub-diffusive or constrained diffusion behavior (Figure 2C) (29). Correspondingly, the D_app_ values dropped as the length of time between captures increased. Average D_app_ values from 2.5 s interval capture experiments were 2.5 ± 2.3 × 10^−^^3 ^µm^2^ s^−^^1^and 1.6 ± 1.4 × 10^−^^3 ^µm^2^ s^−^^1^ for the long and short axis, respectively. The average D_app_ for two dimensions was 2 × 10^−^^3 ^µm^2^ s^−^^1^. This value is similar to that of a 15-kb RK2 derivative, which has been reported to have an average D_app_ of 0.4 × 10^−^^3 ^µm^2^ s^−^^1^ (21). Furthermore, the values are between those reported for GFP and chromosomal loci labeled by FROS, which are 7.7 and 4–5 × 10^−^^5 ^µm^2^ s^−^^1^ (13,30,31) (‘Discussion’ section).

We then determined the area of constraint from the 10s interval MSD plots (Figure 2C and D). A fraction of the spots studied produced values much longer than the dimensions of the cell, suggesting that the duration of the experiment was not long enough for the MSD curve to reach a plateau (Supplementary Figure S2A). From the curves that reached a plateau, we obtained values of 0.43 ± 0.19 and 0.4 ± 0.17 µm (±S.D.) in the long and short axis, respectively. Although the distribution is heterogeneous, in some cases the area approaches the dimensions of the width of the cell, suggesting that these plasmids are constrained by the cell boundaries in the short axis but not in the long axis.

Use of operator arrays did not have any effect on the stability of plasmids but could still result in potential artifacts when studying their diffusion. To corroborate the results described above, similar experiments were done taking advantage of the ∼5-min association of ß-clamp with the plasmid after replication (below) (Supplementary Figure S2B). Using time-lapse experiments with 0.5, 1 and 5 s intervals, we obtained similar results in plasmids labeled with DnaN-YPet, suggesting a constrained behavior with equivalent D_app_ values (from 5-s intervals) of 2.7 ± 1.6 × 10^−^^3 ^µm^2^ s^−^^1^ and 0.9 ± 0.5 × 10^−^^3 ^µm^2^ s^−^^1^ for long and short axis, respectively (Supplementary Figure S2C). The area of confinement was nevertheless smaller, with 0.47 ± 0.21 and 0.28 ± 0.11 µm (±S.D.) in the long and short axis, respectively. The small differences may be due to a change in the morphology or physiology of the cells caused by the different growth conditions used (‘Materials and Methods’ section). Nevertheless, these numbers clearly argue against a model in which plasmids are actively targeted to the poles and fixed to a cellular structure.

### Movement of plasmid mass correlates with the nucleoid reshaping

**Figure 3.**
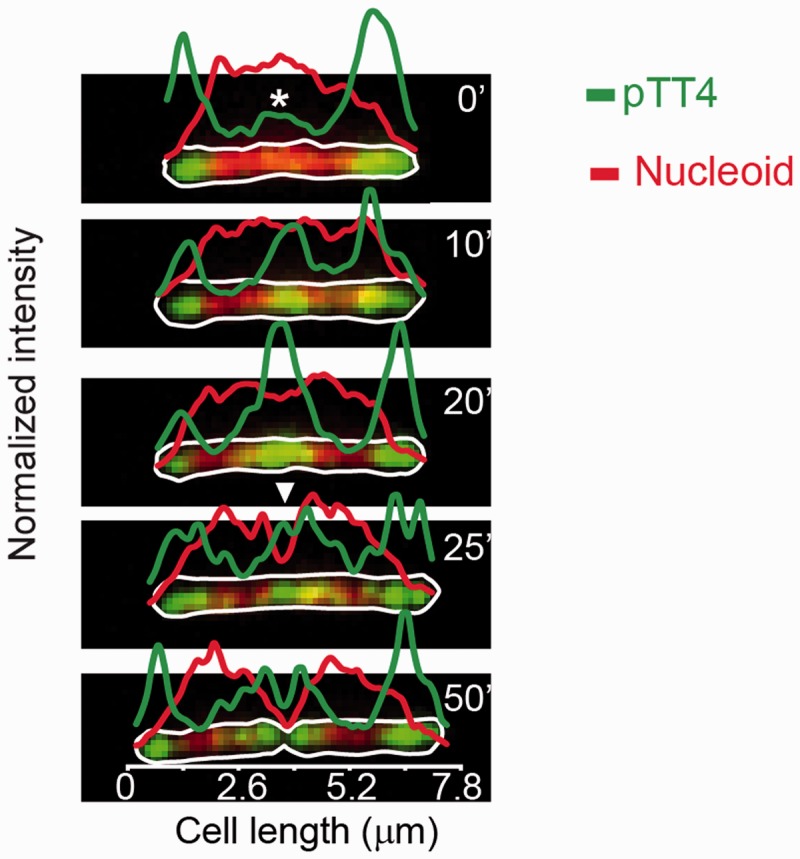
Dynamics of plasmid localization. Pictures of a growing cell showing fluorescent signal of the pTT4 plasmid (green) and the nucleoid (red). The normalized signal distribution of each of the channels is shown as a trace above the corresponding picture. The white star shows the earliest time point at which plasmid signal starts accumulating at midcell, while the white arrow shows the time point of nucleoid splitting. Cell outlines were obtained using phase contrast (white lines). The apostrophe after numbers denotes minutes.

Distribution of plasmids in snapshots and short time-lapse experiments showed that they accumulate at midcell in cells with lengths in the mid-long range of the distribution. Accumulation at midcell in mother cells would result in plasmids localizing at the new poles in daughter cells. Using 5-min interval time-lapse experiments, we observed that this was the case (Figure 3 and Supplementary Figure S3). Profiles of the fluorescence of cells over the cell length clearly show a large redistribution of plasmid molecules in few minutes, often correlating with movement of nucleoids and reiterating the idea that plasmids are constantly moving. Stable accumulation at midcell corresponds to a stage of the cell cycle before clear separation between sister nucleoids and subsequent cell division (Figure 3 and Supplementary Figure S3). These data reiterate the importance of a nucleoid’s shape and position in determining the distribution of plasmids.

### Plasmid replication occurs mainly at the poles throughout the cell cycle

**Figure 4.**
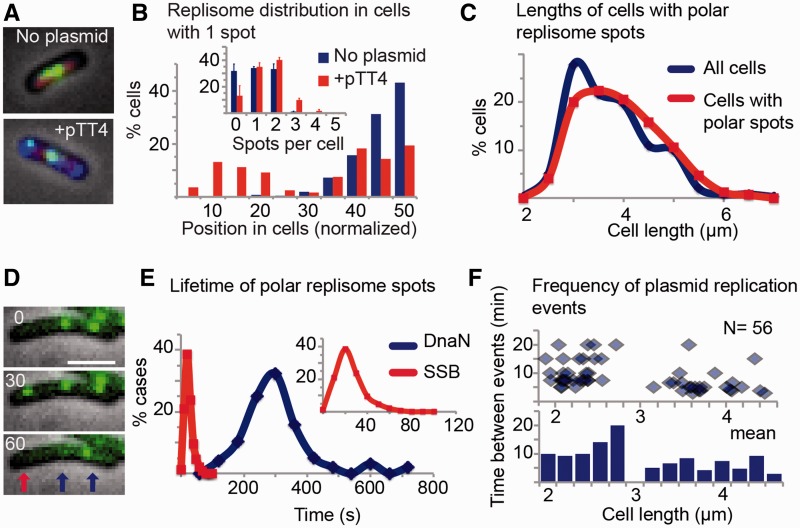
Position and timing of plasmid replication. (**A**) Representative images showing cellular localization of replisomes in cells without (top) and with plasmid (bottom). The nucleoid was labeled with Fis-mCherry (red), replisome with YPet-DnaN (green) and plasmid molecules with TetR-mCerulean (blue). (**B**) Localization frequency of fluorescent spots for cells carrying a single focus. A total of 279 cells not carrying plasmids and 198 cells carrying plasmids were measured to determine the position of replisomes (YPet-DnaN) in the cell. Only half of the cell is represented in the plot for positioning. The inset shows the number of replisome spots as a function of location in the total population. The number of YPet-DnaN spots was determined counting at least thousand cells in each of two independent experiments. Error bars SD. (**C**) Length distribution of all cells (N = 391) (blue) and cells carrying polar replisome spots (N = 170) determined using SSB-YPet (red). (**D**) Representative cell carrying pJHCMW1 labeled with SSB-YPet. A short-lived polar spot (red arrow) appears and disappears within a 30-s time-lapse, while other spots likely corresponding to chromosomal DNA synthesis remain stable (blue arrows). The numbers indicate seconds. (**E**) Frequency of the lifetimes of polar replisome spots determined by measuring SSB-YPet (red, N = 148) and YPet-DnaN foci (blue, N = 96) in cells carrying pJHCMW1. Data obtained from movies at 1-min (YPet-DnaN) or 10-s (SSB-YPet) intervals. The inset shows a graph with the first 120 s expanded. (**F**) Time between events of polar YPet-DnaN spots as a function of cell length in cells not undergoing chromosomal replication. Top panel shows data for individual cells and bottom panel shows binning of the data according to cell length.

The cell’s replisomes are normally positioned between one-fourth to three-fourth regions of the cell length (13). Using DnaN-YPet as a marker, 99 and 87% of the spots lie within this region in plasmid-free cells having one and two spots, respectively (Figure 4A and B and Supplementary Figure S4A). However, this pattern changed in cells with plasmids, polar replisome spots became common and the proportion of spots between one-fourth to three-fourth regions drop to 61 and 69% for cells with one and two spots, respectively. The number of replisome spots per cell increased, resulting in fewer cells without DnaN spots (from 31 to 13%) and an increase of cells with three or more DnaN spots (from 1 to 10%) (Figure 4B). A similar shift toward a polar localization of replisomes was previously reported for *Bacillus subtillis* cells carrying plasmids (32). These data show that the place where plasmid replication occurs is independent of that of the chromosome and often occurs at the cell poles, coinciding with a high density of plasmids in these regions.

Given that both, chromosome and plasmids, use the same machinery to replicate, we wondered if this creates a preference for the timing of plasmid replication during the cell cycle. To test this, we compared the distribution of cell length, a broad indicator of the stage of the cell cycle, between the whole population and cells with polar SSB-YPet (Figure 4C). The cell length distributions were similar, suggesting that replication of plasmids occurs at a similar frequency regardless of the stage of the cell cycle (Figure 4C). Similar results were obtained when DnaQ-YPet was used as replisome marker (Supplementary Figure S4B).

Time-lapse microscopy corroborated the assumption that polar replisome spots mark points of plasmid replication. Regardless of the component used as a marker, most polar spots were shorter-lived than spots close to midcell, likely representing points of chromosome replication (Figure 4D). Such a difference in timing was expected since the extent of DNA to be synthesized is hundreds of times greater in the case of the chromosome. Nevertheless, the lifetime of putative plasmid replisome spots varied depending on the replisome component used. SSB polar spots lasted 24.7 s on average (Figure 4E), a time close to the expected time of synthesis of 11 kb at ∼600 bp s^−^^1^ (13,33). In contrast, polar spots of fluorescent DnaN lasted 302 s on average (Figure 4E), suggesting that sliding clamps remain bound to DNA for ∼5 min after replication ends. Although precise determination of DnaQ spot lifetime was not possible owing to their fast bleaching, qualitative data suggest similar timing to that of SSB. This information agrees with our current model for the dynamics of the replisome, with components having different turnover rates, and with the role of the sliding clamp in processing of DNA after replication (34).

### Plasmids are duplicated in independent events distributed randomly through the cell cycle

Since pJHCMW1 is a ColE1-type plasmid, its replication is initiated in *cis* by a long RNA molecule that serves as primer (RNA II) and a short RNA molecule that acts in *trans* inhibits priming (RNA I) (35). Local and global changes in the concentration of the inhibitor RNA I should determine a preference in the location and timing of plasmid replication. Depending on how fast RNA I diffuses in the cell, it could be predicted that its concentration must be higher where plasmid concentration is higher, i.e. at the cell poles. Plasmids that have escaped from the poles would then have a higher chance to initiate DNA replication. To determine the relationship between plasmid density and replication, we studied the frequency of replication events with respect to their position in the cell. By studying short-lived SSB spots in plasmid-containing cells, we observed that in 83% of the cases, they appeared in a region between the pole and 20% of the cell length (Supplementary Figure S4C). Chromosomal replication, which normally occurs closer to midcell, may mask plasmid replication events happening outside the poles. Nevertheless, data using DnaN-YPet in longer interval time-lapse experiments detected a plasmid replication event every 8.8 min on average (Figure 4F). This number is similar to the 8.3 min needed to replicate 12 plasmids in a 100-min generation time, suggesting that we are able to detect most plasmid replication events. The data with DnaN also show an inverse correlation between cell length and event frequency, resulting in 10.5 and 6.4 min of average delay for cells of 2–2.5 µm and 3–4 µm in length, respectively (Figure 4F). These data suggest that most events occur at the poles and, as a consequence, that replication happens where plasmid density is higher. This further hints to a high enough diffusion rate of RNA I to equalize plasmid initiation potential throughout the cell.

**Figure 5.**
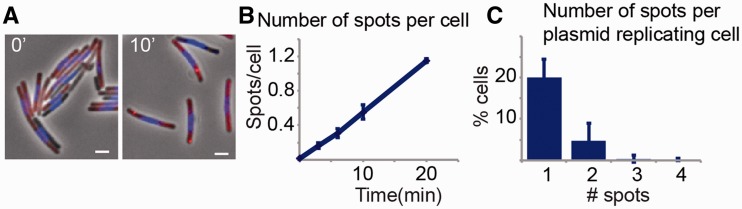
Plasmid replication events. (**A**) Representative pictures of newly replicated DNA in cells carrying a *dnaA*(ts) mutation at nonpermissive temperature. Pictures show cells after 0 and 10 min exposure to EdU (red). Total DNA was stained with DAPI (blue). Plasmid replication is observed as extrachromosomal points of fluorescence after 10 min of labeling. (**B**) Average number of spots per cell after different times of EdU labeling. Data obtained from three independent experiments. At least 300 cells were counted per time point. (**C**) Number of spots in cells carrying at least one spot after 6 min of EdU labeling. Error bars represent SD.

To study the relation between replication events in time in a single cell, we studied plasmid replication by labeling newly synthesized DNA. We used the nucleoside analogue EdU in *dnaA_ts_* cells that in restrictive conditions are able to undergo plasmid but not chromosome replication (36). Plasmid replication events were observed as small fluorescent spots, much dimmer than the labeled chromosome, only found in cells carrying plasmids and only after introducing EdU in the medium for at least 1 min (Figure 5A). The appearance of these spots occurred gradually in the population, but much slower than what it would have been expected if the frequency of plasmid duplication events were as in wild-type cells (Figure 5B). It required 17.6 min of continued exposure to EdU to obtain on average one spot per cell (Figure 5B), indicating that even when plasmid replication continued, the efficiency of initiation dropped. Assays using SSB as fluorescent marker of replication also showed a drop in the number of plasmid replication events. The number of cells with short-lived polar SSB spots at any given time went from 3.5% in the wild type to 1.2% in the mutant. Therefore, it is most probable that the reduction observed is not due to a low efficiency of EdU incorporation. This reduction in efficiency could be due to the loss of an unknown enhancer effect of DnaA on the initiation of plasmid replication, although we could not find a plasmid-encoded DnaA box. Similar results were observed with ColE1, but in this case, a DnaA box was found (7). An alternative possibility is that loss of DnaA results in an indirect detrimental effect on replication. Using a short EdU labeling period of 6 min, we found that single-plasmid replication events per cell are 4 and 50 times more likely to happen than events with two and three replications, respectively (Figure 5C). Thus, replication events seem to occur independently for each plasmid in the cell.

## DISCUSSION

Reports on partition of ColE1-type plasmids and derivatives have raised the possibility that plasmid molecules group in clusters at the poles within cells (7,9,10), in contrast to previous models proposing that they diffuse freely in the cytoplasm (37). Although no explanation for the nature of the clusters or how they are held together is available, models to explain these facts have been attempted (38). Here we present experimental evidence that reconciles both models using pJHMCW1. Plasmids were mainly found at the poles, but this localization is due to displacement of the plasmid molecules by the nucleoid rather than to the presence of an uncharacterized partition system. Our data demonstrate that plasmids can freely diffuse inside the nucleoid-free regions at the poles and occasionally move out of them, migrating through the nucleoid-dense region before getting trapped in another or the same nucleoid-free region. A similar model has been proposed to explain diffusion of membrane proteins through different membrane compartments by Hop Diffusion (39). Confinement of most plasmid molecules to the nucleoid-free poles ensures efficient transfer to the two daughter cells while restricting the number of freely diffusing plasmids that can potentially end up in either half after division. Any stable clustering would require physical plasmid–plasmid or plasmid–cell interactions that restrict movement and maintain molecules together, with the consequence that small numbers of clusters could not be partitioned randomly if they were to be stably inherited over generations (40).

We calculated the diameter of gyration of supercoiled pJHCMW1 molecules as 265 nm, which is significantly bigger than that of the ribosome, 18 nm (24), which has been shown to be large enough to be excluded by the nucleoid (41,42). Nucleoid exclusion of hcn plasmids that do not encode partition systems is also supported by their presence in minicells (7,43). We propose that by limiting the free movement of the plasmid molecules (21), partition systems interfere with their tendency to occupy the nucleoid-free regions. For example, the ParAB-*parS* partition systems for low copy plasmids use the chromosome as a matrix to facilitate partition (44). Localization to nucleoid-free regions has been observed for F, R1, RK2 and P1 derivatives lacking functional partition systems (8,45–49). Furthermore, a 15-kb RK2 derivative that lacks the partition system diffuses in a similar way to pJHCMW1 moving at an average step size of 0.04 ± 0.024 µm s^−^^1^, which is close to the 0.071 ± 0.06 µm s^−^^1^ observed at 5-s interval in our experiments (21). However, unlike hcn plasmids, these derivatives fail to be efficiently segregated most probably owing to their low copy number.

It has long been established that some plasmids replicate at random within a population, causing individual molecules to replicate more than once and some not to replicate during a cell generation (50,51). We now have established that replication occurs throughout the cell cycle. At a similar plasmid density, longer cells have more replication events owing to the higher number of molecules. A similar conclusion was reached in a study using mini-F and pBR322 derivatives and the baby-machine technique (52). Replication seems to occur most frequently at the poles, coinciding with a higher density of plasmids (Supplementary Figure S4B). A distinct replisome distribution was also observed in *B. subtillis* and *Caulobacter crescentus* cells carrying plasmids, suggesting they were localized to sites normally occupied by plasmids (32,53). Although some RNA species may be constrained to the regions close to transcription, particularly those transcripts that encode translationally coupled proteins (54), our data suggest that RNA I molecules freely diffuse throughout the cell because we did not observe a replicating advantage for the plasmids that escape the poles. As a consequence, RNA I, estimated to have an average copy number of ∼400 molecules per cell (55), is present at a sufficiently high level throughout the cell to have a continuous repressing activity on the plasmids, therefore being responsible for mediating incompatibility between related hcn plasmids. Future studies measuring the RNA I concentration at different locations of the cell will permit us to test this hypothesis. In this model, the probability of a plasmid initiating replication does not depend on its position within the cell and instead each has a similar probability to be replicated. At constant RNA I levels, what determines the rate of replication initiation is the frequency at which RNA II becomes a productive primer.

We think that the stringency of the nucleoid to act as a barrier will depend on the size of the plasmid, likely contributing to the large plasmids need for partition systems (56). Big molecular assemblies that also require active systems for partition, like carboxysomes or chemotaxis protein clusters (57,58), may do so not only to generate evenly spaced patterns, but also to withstand the tendency to be displaced to the poles as in the case of inclusion bodies (59).

A growing number of ColE1-type plasmids are being found that carry genetic determinants for resistance to clinically important antibiotics and participate in their dissemination (11,60–64). A deep understanding of their maintenance functions and dissemination is necessary to help designing strategies to preserve our ability to combat bacterial infectious diseases. For example, methodologies that enhance the negative control of RNA I or the ability to move to the newly formed nucleoid-free spaces in the later stages of the cell cycle could destabilize ColE1-type plasmids, leading to loss of acquired antibiotic resistance and limiting its dissemination.

## SUPPLEMENTARY DATA

Supplementary Data are available at NAR Online.

## FUNDING

National Institutes of Health Public Health Service Grant [2R15AI047115 to M.E.T.]; Wellcome Trust Programme [WT083469MA to D.J.S.]; Wellcome Trust Strategic Award [091911], supporting advanced microscopy at Micron Oxford (http://micronoxford.com); Todd-Bird Fellowship at New College, Oxford [to R.R.L.]; LA Basin Minority Health and Health Disparities International Research Training Program (MHIRT) [5T37MD001368 to T.T., D.M., L.L. and A.M.L.]; National Institute on Minority Health and Health Disparities, NIH. Funding for open access charge: National Institutes of Health.

*Conflict of interest statement*. None declared.

## Supplementary Material

Supplementary Data
