# Supplementary material for: High-copy bacterial plasmids diffuse in the nucleoid-free space, replicate stochastically and are randomly partitioned at cell division
Source: Nucleic Acids Res. 2013 Oct 16;42(2):1042–51. doi: 10.1093/nar/gkt918 (PMC3902917; doi:10.1093/nar/gkt918)
Supplement: Supplementary Data [file supp_42_2_1042__index.html]

High-copy bacterial plasmids diffuse in the nucleoid-free space, replicate stochastically and are randomly partitioned at cell division — Supplementary Data 

# High-copy bacterial plasmids diffuse in the nucleoid-free space, replicate stochastically and are randomly partitioned at cell division

## Supplementary Data

files

**Files in this Data Supplement:**

- Supplementary Data - pdf file
- Supplementary Data - mov file
