# Supplementary material for: High-copy bacterial plasmids diffuse in the nucleoid-free space, replicate stochastically and are randomly partitioned at cell division
Source: Nucleic Acids Res. 2013 Oct 16;42(2):1042–51. doi: 10.1093/nar/gkt918 (PMC3902917; doi:10.1093/nar/gkt918)
Supplement: Supplementary Data [file supp_gkt918_nar-02209-h-2013-File007.pdf]

High-copy bacterial plasmids diffuse in the nucleoid-free space, replicate stochastically and are randomly partitioned at cell division

Rodrigo Reyes-Lamothe[1,2] *, Tung Tran[1,3], Diane Meas[1], Laura Lee[1], Alice M. Li[1], David J. Sherratt[1] and Marcelo E. Tolmasky[1,3]

[1]Department of Biochemistry, University of Oxford, Oxford OX1 3QU, UK
[2]Department of Biology, McGill University, Montreal, Quebec H3G 0B1, Canada
[3]Center for Applied Biotechnology Studies, Department of Biological Science, College of Natural Science and Mathematics, California State University Fullerton, Fullerton, California 92834-6850

* To whom correspondence should be addressed. Tel: 1 (514) 398-5137; Fax: 1 (514) 398-5069; E-mail: rodrigo.reyes@mcgill.ca

SUPPORTING INFORMATION

Figures S1-S4

Video S1

SUPPORTING FIGURES

Figure S1. Fluorescence labeling and localization of plasmids in elongated cells. (A) Map of pJHCMW1 plasmid showing its most relevant features. (B) Diagram of the system used to label plasmid molecules. Fluorescent TetR repressor expressed from a gene inserted in the chromosome binds to its cognate operator in the plasmid. Plasmids pTT3 and pTT4 carry either 48 or 96 operators respectively. (C) Stability of the plasmid was tested by growing cells in liquid medium without selection and spotting dilutions after 60 generations on plates with or without ampicillin in the presence or absence of IPTG (upper panel). Bottom panel shows the level of fluorescence of unselected cells with continuous expression of the repressor after 1h (less than one generation) or 70 generations. (D) Plasmid molecules (red) spread over nucleoid-free regions in cells carrying a conditional replication mutation at the non-permissive temperature. (E) Plasmid molecules (red) fill spaces between nucleoids (blue) in cells treated with the cell division inhibitor cephalexin.

Figure S2. Movement of plasmid molecules. (A) Level of constrained plasmids, as obtained from individual Mean Square Displacements (MSD), showing those that stay within cell boundaries (circled in red). Those molecules within the cell boundaries are shown in greater detail in Fig. 2*D*. There are more plasmids seemingly without constraint in the long axis than in the short axis. (B) Time lapse using 1.5 second intervals of a cell containing two YPet-DnaN spots. Red arrow points at a brighter, centrally positioned spot that likely marks replication of chromosome. Blue arrow shows a more mobile, dimmer replisome spot that likely represent plasmid replication. (C) MSD and Apparent Diffusion rate (D_{app}) plots of traces obtained tracking Ypet-DnaN spots at 0.5 (N=30), 1 (N=28) and 5 (N=21) second intervals. Error bars represent SD. (D) Projected area of constraint in the cell of individual plasmid molecules studied at 5 second intervals obtained from their MSD curves.

Figure S3. Dynamics of plasmid localization. Pictures of the same growing cell over time showing fluorescent signal corresponding to plasmid (green) and nucleoid (red). The normalized signal distribution of each of the channels is shown as a trace above the

corresponding picture. The data for three other independent cells is shown as traces without pictures. In cell #1 the white star shows the earliest time-point at which plasmid signal starts accumulating at the midcell, while the white arrow shows the point of clear nucleoid splitting. Cell outlines were obtained from phase contrast (white lines).

Figure S4. Plasmid and replisome localization. (A) Localization of replisomes in cells carrying two foci. The position YPet-DnaN inside the cells was determined in plasmidless cells (N=241) and plasmid-carrying cells (N=199). Error bars SD. (B) Length distribution of cells carrying polar replisomes, identified by labeling with DnaQ-YPet (N=272), compared to the total population (N=981). The data was obtained from two independent experiments. Error bars SD. (C) Position of short-lived SSB-YPet spots in plasmid carrying cells. The plot shows the position of the spots on the normalized length of the cells with respect to the cell length. Inset shows the frequency of binned data irrespective of the length of cells used for the study.

SUPPORTING VIDEO

Video S1. Short time-lapse of a cell carrying pTT3 labeled with TetR-YPet. Interval between pictures is 4s. A spot splits and moves from midcell to a region close to one of the poles while the fluorescence of plasmids at the poles shifts continuously.

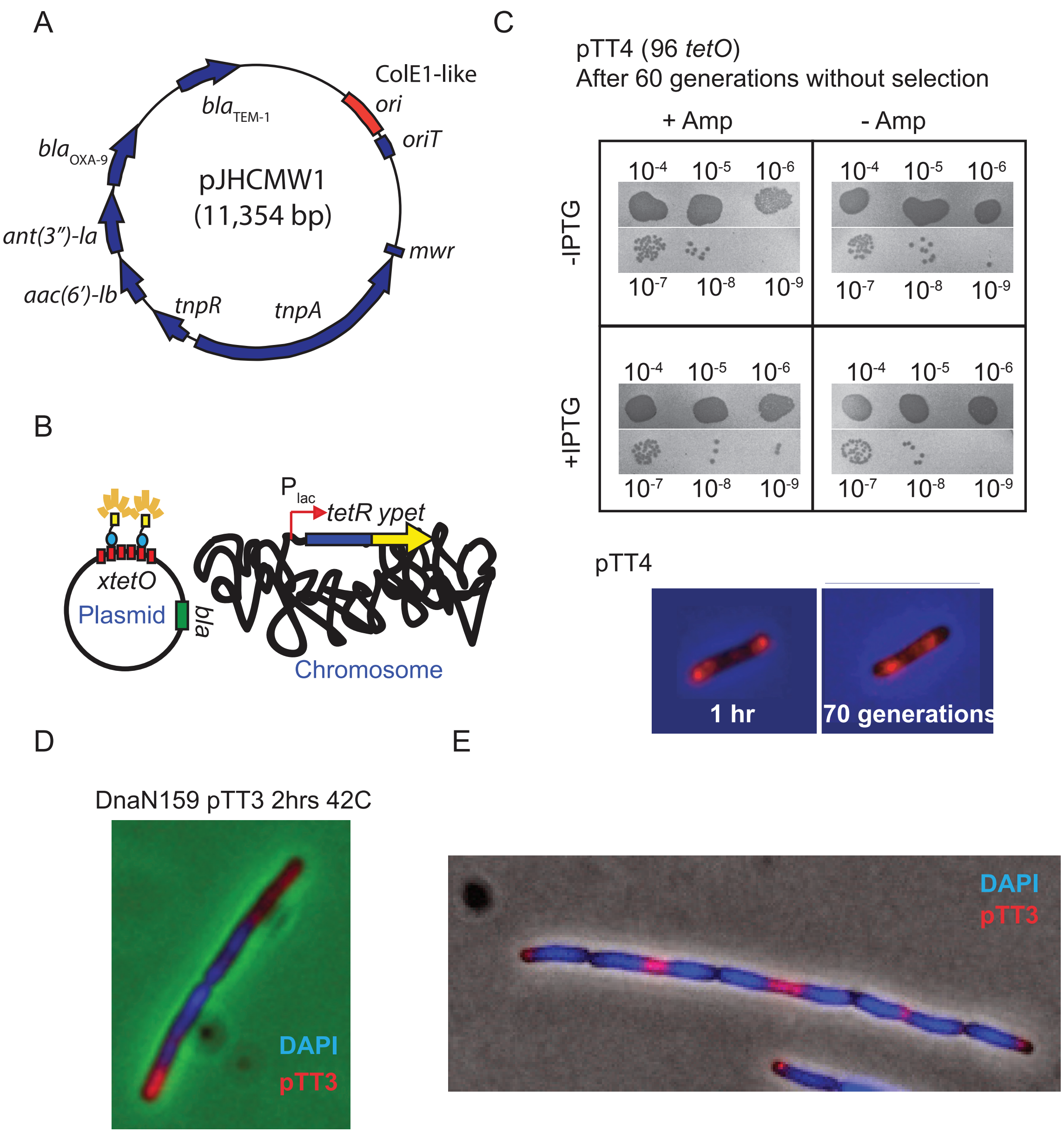
A
C
pJHCMW1
(11,354 bp)
bla TEM-1
ColE1-like
ori
oriT
mwr
tnpA
tnpR
aac(6')-Ib
ant(3")-Ia
bla OXA-9
pTT4 (96 tetO)
After 60 generations without selection
+ Amp
- Amp
-IPTG
+IPTG
10-4 10-5 10-6
10-7 10-8 10-9
B
P lac
tetR ypet
xtetO
Plasmid
bla
Chromosome
pTT4
1 hr
70 generations
D
E
DnaN159 pTT3 2hrs 42C
DAPI
pTT3

Figure S1_

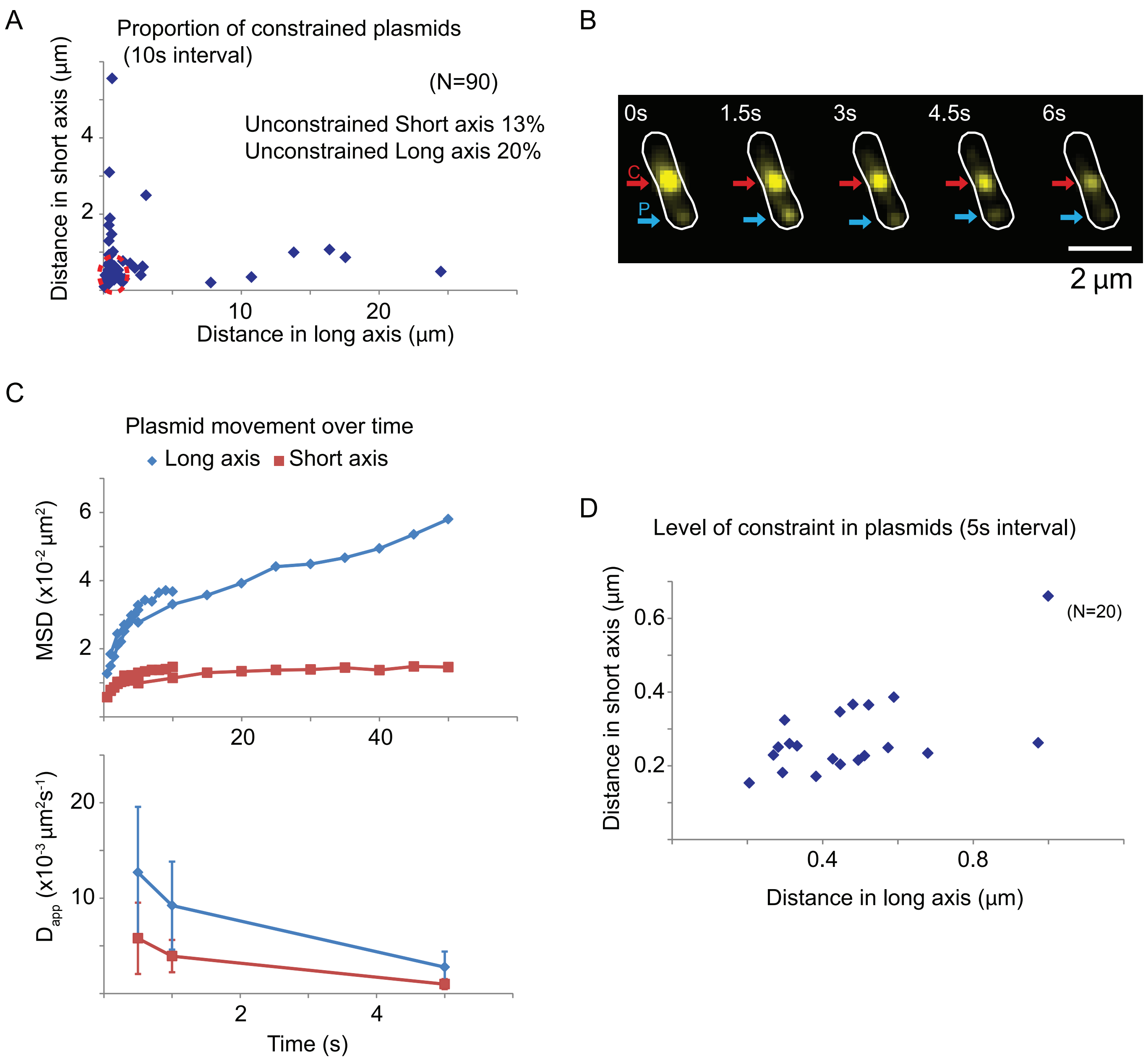
A
Proportion of constrained plasmids (10s interval)
(N=90)
Unconstrained Short axis 13%
Unconstrained Long axis 20%
Distance in short axis (μm)
Distance in long axis (μm)
B
0s
1.5s
3s
4.5s
6s
C
P
2 μm
C
Plasmid movement over time
Long axis
Short axis
MSD (x10-2 μm2)
Dapp (x10-3 μm2s-1)
Time (s)
D
Level of constraint in plasmids (5s interval)
(N=20)
Distance in short axis (μm)
Distance in long axis (μm)

Figure S2_

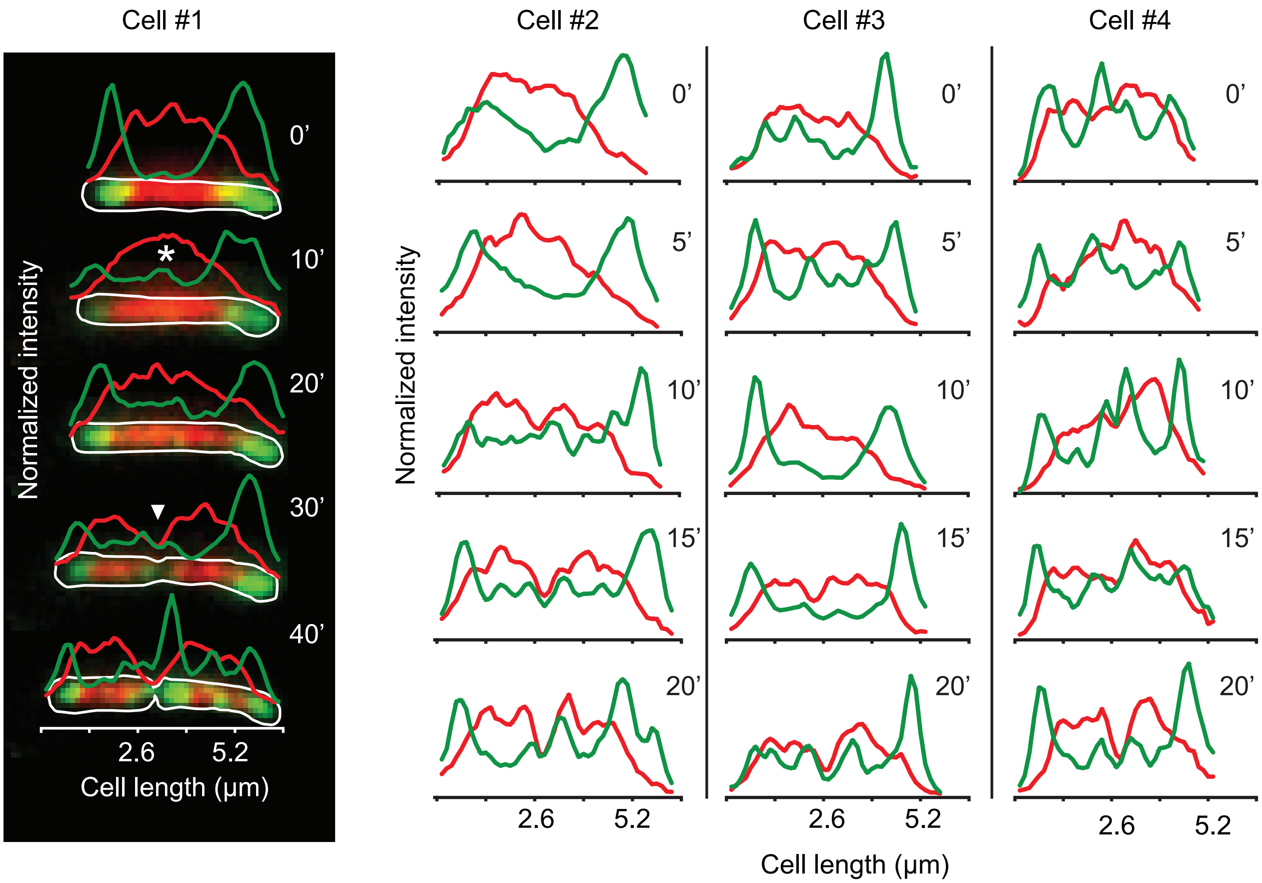
Cell #1
Cell #2
Cell #3
Cell #4
0'
10'
20'
30'
40'
5'
15'
20'
Normalized intensity
2.6
5.2
Cell length (µm)

Figure S3_

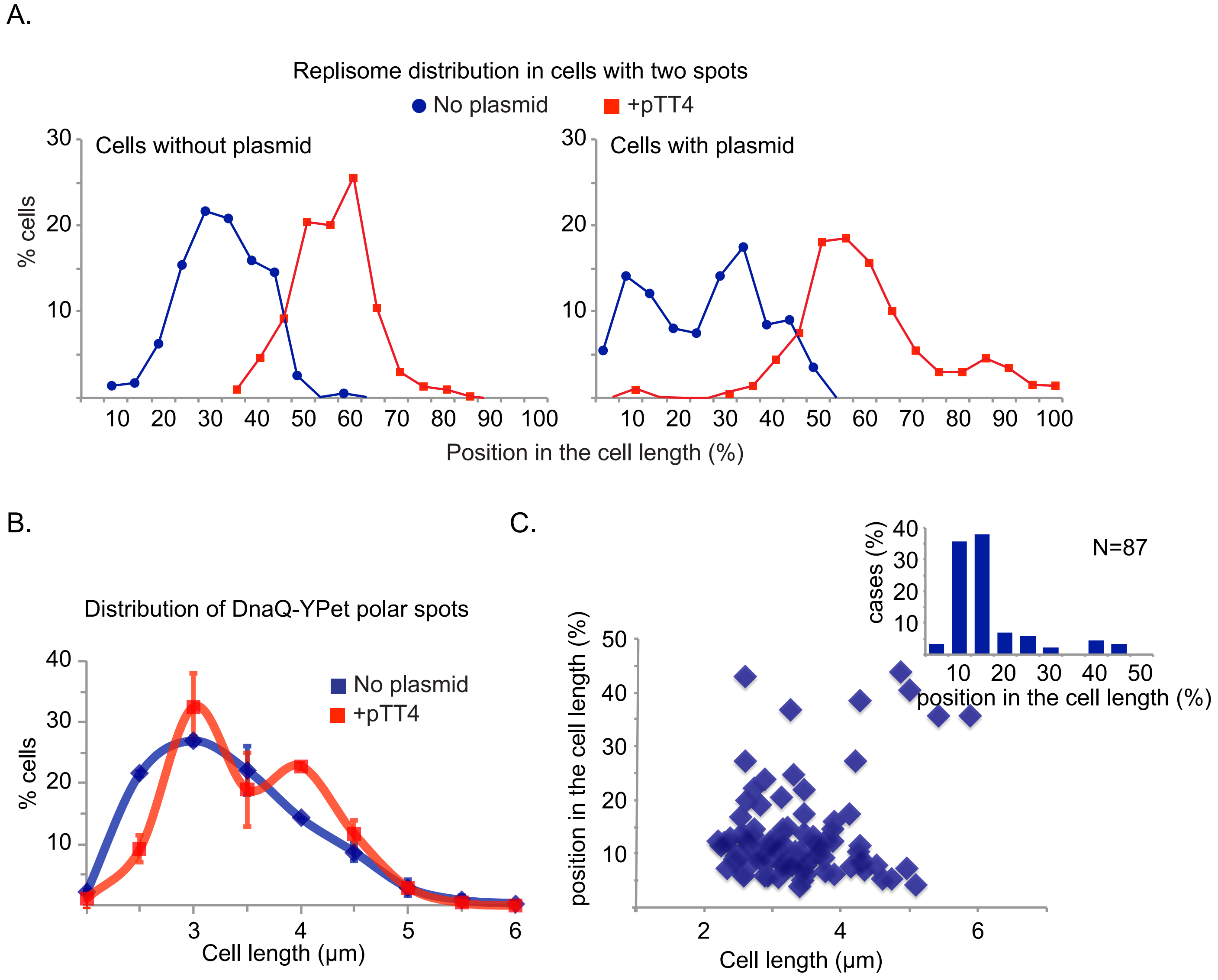

A.
Replisome distribution in cells with two spots
No plasmid
+pTT4
Cells without plasmid
Cells with plasmid
% cells
Position in the cell length (%)
B.
Distribution of DnaQ-YPet polar spots
No plasmid
+pTT4
% cells
Cell length (µm)
C.
N=87
cases (%)
position in the cell length (%)
position in the cell length (%)
Cell length (µm)

Figure S4_
